# The Effect of Green Synthesized CuO Nanoparticles on Callogenesis and Regeneration of *Oryza sativa* L.

**DOI:** 10.3389/fpls.2016.01330

**Published:** 2016-08-31

**Authors:** Sadaf Anwaar, Qaisar Maqbool, Nyla Jabeen, Mudassar Nazar, Fazal Abbas, Bushra Nawaz, Talib Hussain, Syed Z. Hussain

**Affiliations:** ^1^Department of Biotechnology and Bioinformatics, International Islamic University IslamabadIslamabad, Pakistan; ^2^Department of Biotechnology, Virtual University of PakistanLahore, Pakistan; ^3^Interdisciplinary Research Organization, University of ChakwalChakwal, Pakistan; ^4^Department of Physics, International Islamic University IslamabadIslamabad, Pakistan; ^5^Department of Mechatronics, University of Engineering and Technology TaxilaChakwal, Pakistan; ^6^National Institute of Vacuum Science and TechnologyIslamabad, Pakistan; ^7^Department of Biological Sciences, Quaid-i-Azam UniversityIslamabad, Pakistan

**Keywords:** copper oxide nanoparticles, SEM, XRD, FTIR, callus induction, regeneration

## Abstract

In this study, we have investigated the effect of copper oxide nanoparticles (CuO-NPs) on callogenesis and regeneration of *Oryza sativa* L (Super Basmati, Basmati 2000, Basmati 370, and Basmati 385). In this regard, CuO-NPs have been bio-synthesized via *Azadirachta indica* leaf extract. Scanning electron microscope (SEM) analysis depicts average particle size of 40 ± 5 nm with highly homogenous and spherical morphology. X-ray diffraction (XRD) and Fourier transform infrared spectroscopy (FTIR) have been employed to confirm the phase purity of the synthesized NPs. It is found that CuO-NPs exhibit very promising results against callus induction. It is attributed to the fact that green synthesized CuO-NPs at optimum dosage possess very supportive effects on plant growth parameters. In contrast to callogenesis, differential regeneration pattern has been observed against all of the examined *O. sativa* L. indigenous verities. Overall observation concludes that CuO, being one of the essential plant nutrients, has greatly tailored the nutritive properties at nano-scale.

## Introduction

Plant tissue culture is of prime importance due to its novel and versatile applications in basic and advanced biotechnological research. In commercial point of view, it not only provides genetically modified plants but also responsible for the mass propagation of plants with desired characteristics (Mahna et al., 2013). In plant tissue culture aseptic condition is one of the basic requirement. Microbial contamination in tissue cultured plants is one of the major hurdle which leads to the massive loss of platelets. In plant tissue culture, several reports of silver (Abdi et al., 2008) and titanium oxide (Shiraishi et al., 2009) NPs as antimicrobial agents have been reported.

Callus induction and shoot regeneration are important in cell and tissue culture techniques for plant improvement (Kumar et al., 2009). A valuable export commodity of Pakistan is “Basmati rice.” Many environmental constrains limit its production. In order to meet food requirement worldwide, it is a big need to improve the existing germplasm of Basmati cultivars (Naqvi et al., 2005). Applications of *in vitro* transfer techniques to plant improvement depend on regeneration capacity (Higuchi and Maeda, 1990). Callus cultures are particularly significant in plant biotechnology. Management of the auxin to cytokinin ratio in the medium can be directed to the development of shoots, roots and somatic embryos from which whole plant can subsequently be produced (Tariq et al., 2008).

In modern material sciences, research on nanoparticles (NPs) is one of the most attractive and active areas of research. NPs have wide applications due to their size, structure, and physiochemical properties in industries and agriculture (Safavi, 2014). There are several methods reported for the fabrication of metallic NPs, plants seem to be the best choice, and NPs produced by plants are more stable, least toxic and biocompatible (Iravani, 2011). Toxic effects of NPs for plants and animals have been reported but there is no report till now documented which showed the harmful effects of NPs on tissue culture plants (Zafar et al., 2016). Among all reported NPs so far, CuO-NPs being a significant kind of metal oxide NPs, are widely used in catalysis, biomedical applications, gas sensors, solar energy conversion, high-temperature superconductors, and field emission emitters with unique features and higher efficiency (Yin et al., 2005). Conventionally, Cu in its bulk form is vastly utilized as nutritive plant medium in callus induction and regeneration but lesser specific surface area and lower surface energy limit its reactivity.

Keeping in view, we have followed a facile green synthesis route for CuO-NPs fabrication and its callogenesis and regeneration effects have been examined against important recalcitrant *O. sativa* L. cultivars (Basmati 2000, Basmati 385, Basmati 370, and Super Basmati). So far, to the best of our knowledge, no or very few reports are available in literature for bio-synthesis mediated callogenesis and regeneration effect of CuO-NPs against *O. sativa* L. or any other cultivars. The aim of this study is to elaborate and understand the least toxic and nutritive effects of photo-synthesized CuO-NPs in callus induction and regeneration of *O. sativa* L. It is strongly believed that this work would open new corridors in the field of tissue culture technology.

## Experimental procedures

### Green synthesis of CuO NPs through *A. indica* leaf extract

Authenticated *A. indica* leaves were collected from National Agricultural Research Centre (NARC), Pakistan. For purified extract, 20 g of dried plant leaves were finely grinded and added in 200 mL of deionized H_2_O. This mixture were then placed in shaking incubator for 2.5 h at 40°C at 50 rpm and later on was filtered using Whatman No. 1 filter paper. 7.98 g of Cu(CO_2_CH_3_)_2_ ·H_2_O (Sigma-Aldrich) was added in 200 mL of the leaf extract and put at Scilogex Magnetic Stirrer attuned to 55°Cat ~1500 rpm for ~2.5 h. Thereafter, mixture was collected and repeatedly loaded in GR BioTek centrifuge at ~10,000 rpm for 10 min, brown pellets were isolated during concentration and collected. To remove uncoordinated compounds, washing with deionized water was done thrice. CuO-NPs collected was dried using Memmert Hot Air Oven at 60°C for ~5 h and further calcinations at high temperature was operated in Gallenkamp Furnace at 400°C for ~2.5 h.

### Characterization of biosynthesized CuO-NPs

To observe CuO-NPs morphology, SEM analysis was performed using JOEL_JSM_6490LASEM operating at 20 kV coupled with Energy Dispersive X-ray (EDX) spectroscopy. SEM analysis was carried out by Gold coating CuO-NPs. Crystallographic studies of photosynthesized CuO-NPs was achieved using Cu Kα radiation (λ (Wavelength) = 1.54060 Å) with nickel monochromator in the range of 2θ between 20° and 80° with step size 0.2°. Scherrer's formula (Equation 1) is employed to calculate average crystallite size. Moreover, FT-IR spectroscopy of CuO-NPs were carried out using KBr pellet technique (SHIMADZU_FT-IR) wave number ranging 400–4000 cm^−1^.

### Collection of plant material

Four seed varieties of rice (*Oryza sativa* L.) were obtained from NARC (National Agriculture Research Centre) including Super Basmati, Basmati 2000, Basmati 370, and Basmati 385. The explants were dehusked and surface sterilized with 70% Ethanol. N6 was used as basal medium with 3% Sucrose and 8 g/L Gelrite Agar and pH was adjusted to 5.8 and autoclaved at 121°C for 20 min at 15 psi. After autoclaving the media, CuO-NPs were also added at various mg/L concentrations. To avoid cluster formation of NPs at the base, the medium was allowed to cool up to 45°C and after this the flasks were kept at 4°C till the media solidified.

### Callus induction

For callus induction N6 (Songstad et al., 1991) media (2 mg/L of 2,4-D) was used and supplemented with CuO-NPs (1–20 mg/L) and without CuO-NPs as control. The culture tubes were kept in both dark as well as light (16/8 h photoperiod) conditions at 25 ± 2°C for 12 days. After the specific incubation period of 10–13 days, embryogenic-calli induced from seed scutellum were obtained. Callus induction frequency was obtained by following formula:

$$\begin{array}{l} Callus\;induction\;frequency\left(\%\right)\\ \;=\frac{No.\;of\;calli\;produced\;by\;seeds}{No.\;of\;seeds\;inoculated}\;\times \;100 \end{array}$$

### Regeneration of callus

The embryogenic-calli produced were transferred on three different regeneration media with different concentrations and combinations of hormones supplemented with CuO-NPS and without CuO-NPs as control were tested for best regeneration media combination. After 8–10 weeks regeneration frequency was recorded using the formula given below:

$$\begin{array}{l} Plant\;regeneration\;frequency\left(\%\right)\\ \;=\frac{No.\;of\;calli\;regenerated\;into\;plantlets}{No.\;of\;calli\;inoculated\;for\;regeneration}\;\times \;100 \end{array}$$

### Statistical analysis

Statistical analysis of the data was performed by two factorial Analysis of variance (Statistix 8.1 software). Each experiment was replicated thrice and each replication has 50 observations.

## Results

### Characterization of prepared CuO-NPs

**Figure 1:**
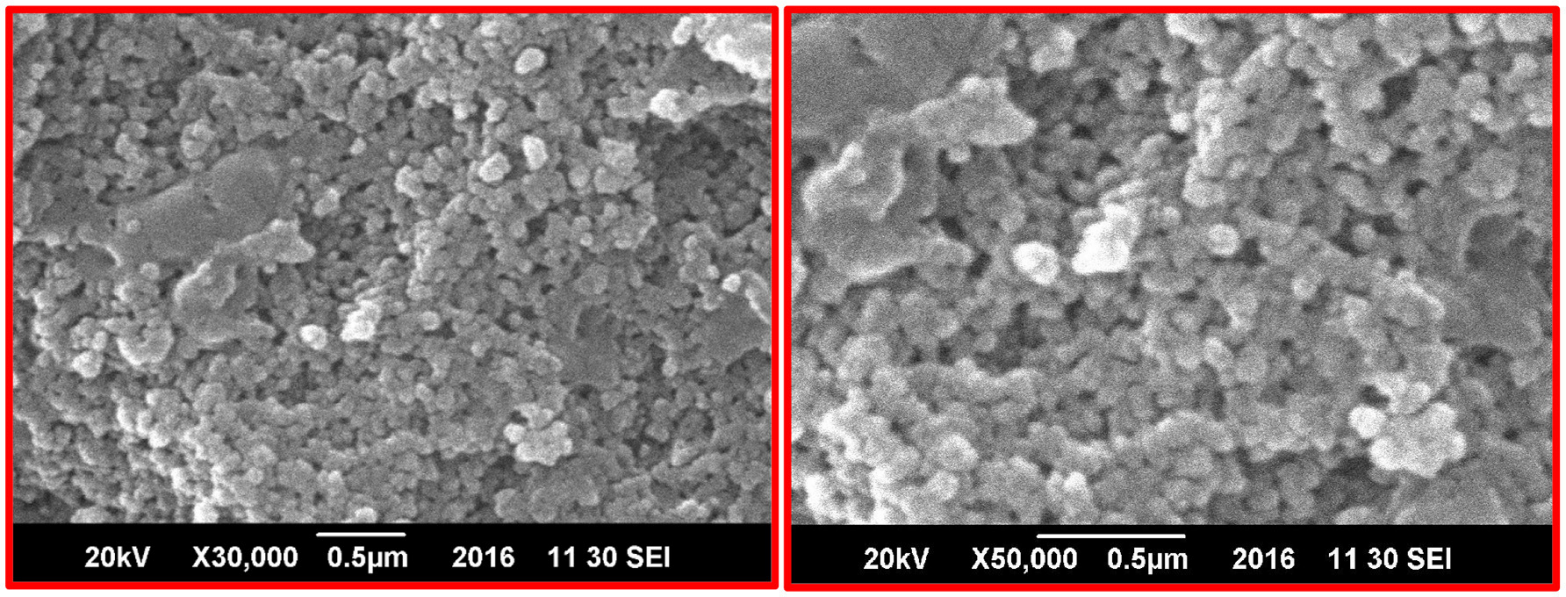
**SEM images of green fabricated CuO-NPs**.

**Figure 2:**
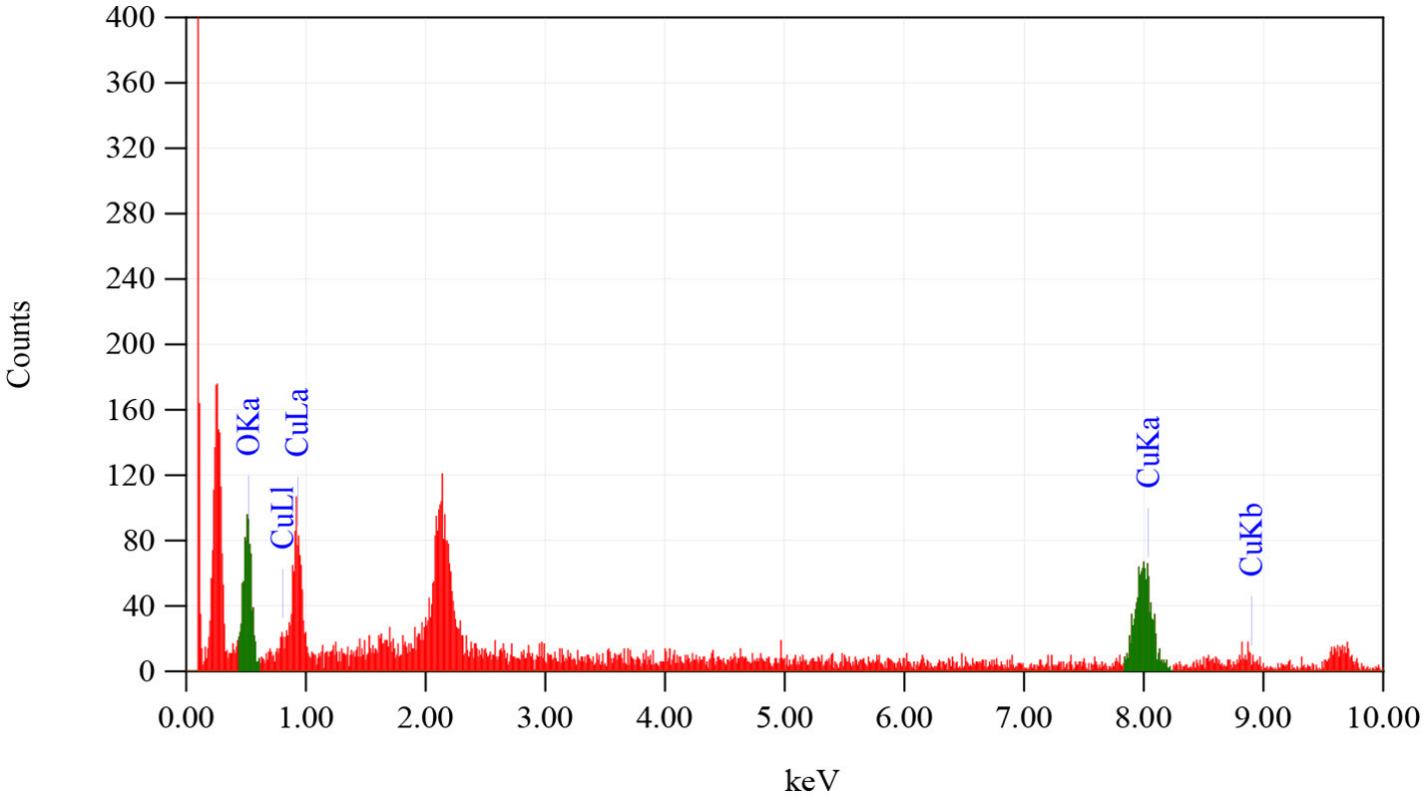
**EDX analysis of CuO-NPs**.

From the SEM analysis, as shown in Figure 1, it is revealed that CuO-NPs synthesized through green chemistry are highly homogenous and spherical in shape having mean particle size of 40 ± 5 nm. EDX pattern in Figure 2 explores us elemental percentage (i.e., by mass 65.33% Cu and 34.67% O) of biosynthesized CuO-NPs. EDX results confirmed that only Cu and O ions are present in the synthesized NPs with the same ratio proportion as defined at the time of experiment.

**Figure 3:**
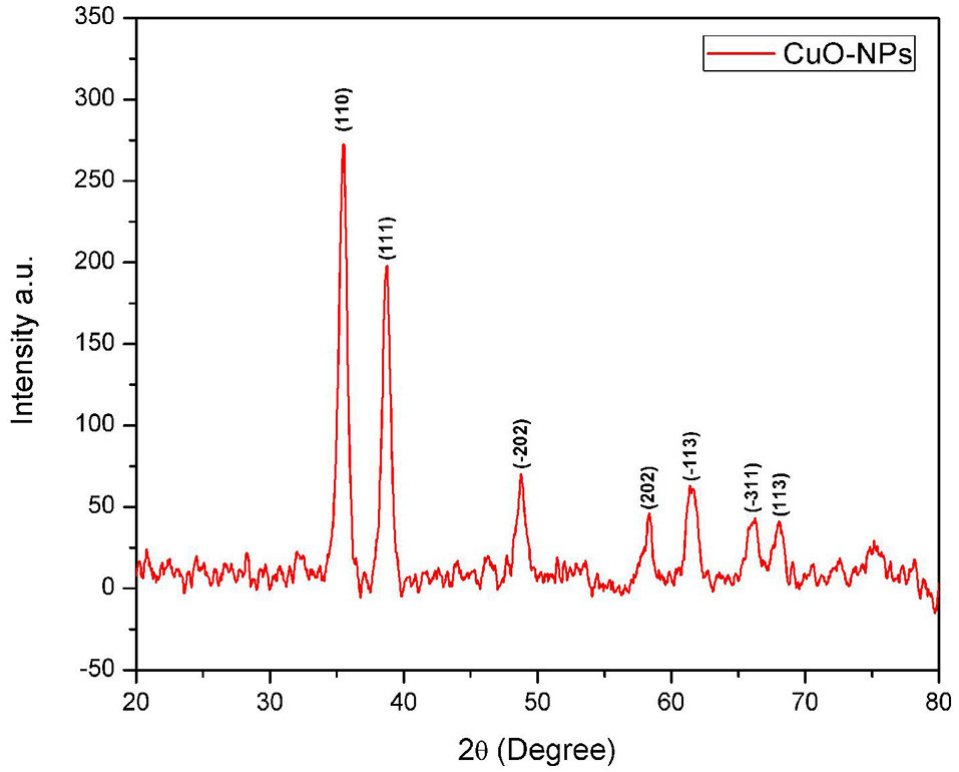
**XRD spectrum of CuO-NPs**.

In order to investigate the phase purity and average crystallite size of the synthesized sample, XRD technique has been utilized. Figure 3 illustrating crystallographic studies of biosynthesized CuO-NPs in the 2θ ranges from 20° to 80° with step size 0.2°. Several prominent Bragg peaks, (110), (111), (−202), (202), (−113), (−311), and (113) are observed in the XRD pattern. Micro structural crystallite size is calculated using Debye Scherrer formula (Equation 1).

(1) (t=0.89 λ/ β cosθ)

Where *t* is the crystallite size, λ is the wavelength of incident Cu-Kα x-rays and β is the full width at half maximum, in radians, of the selected peak (111) at diffraction angle θ. It has been calibrated that average crystallite size is closer to 7 nm.

**Figure 4:**
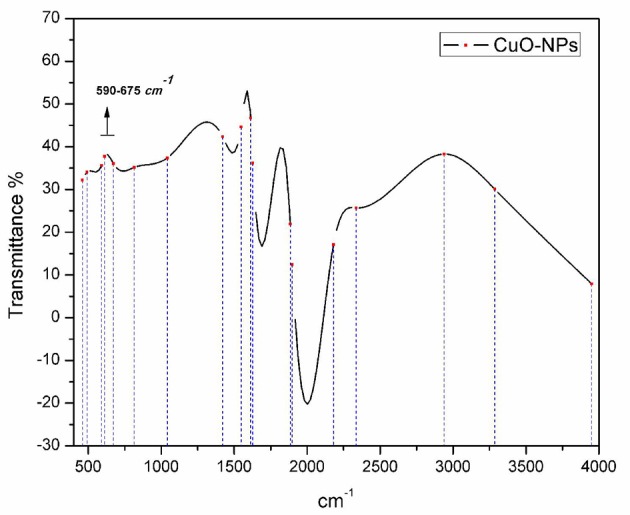
**FTIR spectrum analysis of bio-fabricated CuO-NPs**.

Utilizing non-destructive technique (FTIR spectroscopy) to probe the structural confirmation of molecules present inside the prepared sample, it is found that FTIR spectrum (Figure 4) exhibited Cu-O specified peaks in the range from 590 to 675 cm^−1^. Moreover, OH^−^ and C-O stretching vibrations can be seen from 2800 to 4000 cm^−1^.

### Effect of different concentrations of CuO NPs on N6 medium for frequency of callus induction

**Figure 5:**
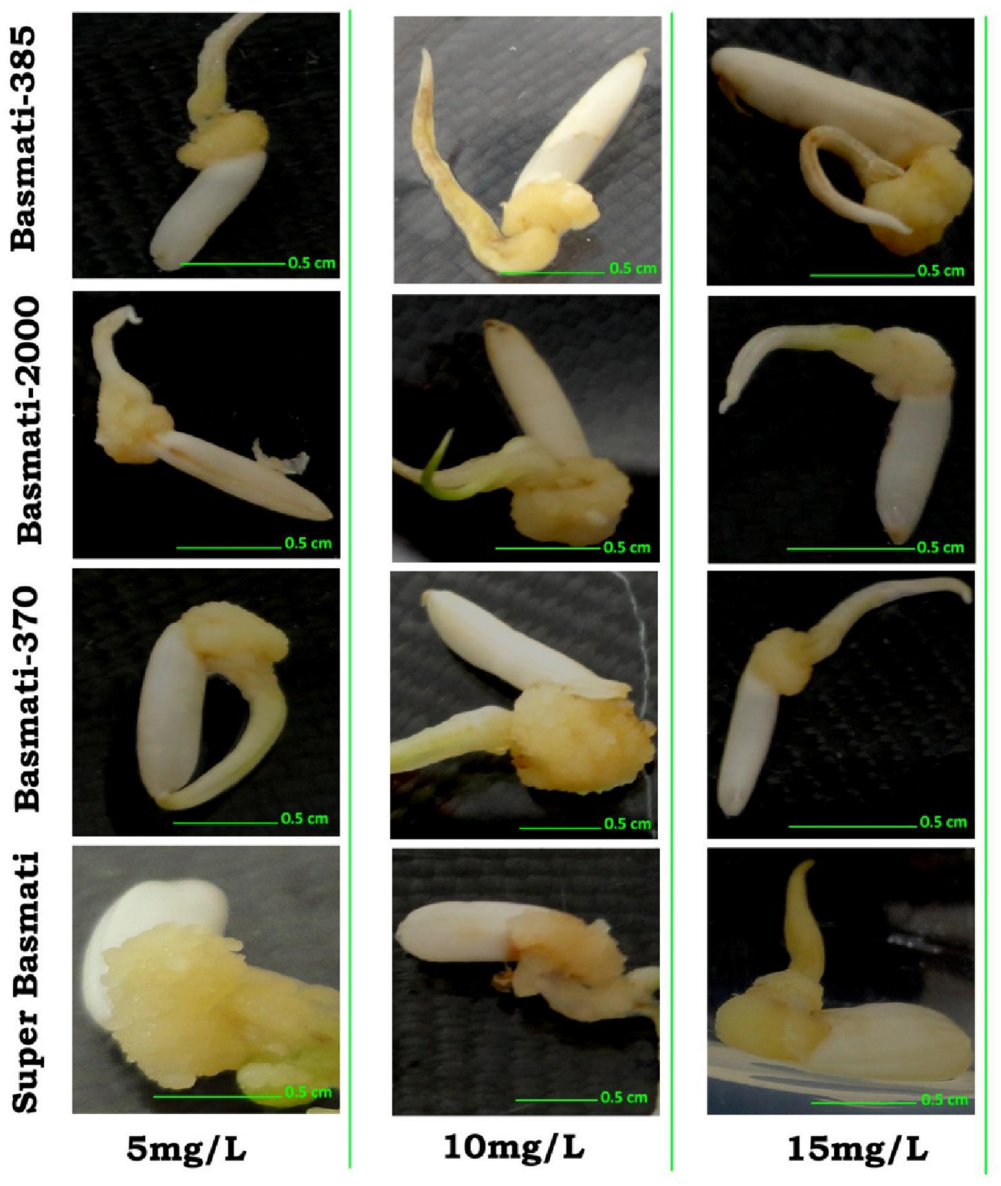
**Effect of CuO-NPs (5, 10, and 15 mg/L) in callogenesis of ***O. sativa*** L. (indeginous verities = Basmati 385, Basmati 2000, Basmati 370, and Super Basmati)**.

Results of different concentrations of CuO-NPs on callogenesis of four Pakistani rice cultivars are documented in Table 1. Results showed that seeds explants significantly responded to the addition of CuO-NPs in the medium. Figure 5 shows response of different concentrations on CuO-NPs on all the tested cultivars. With the increase in concentrations of NPs the callus induction frequency increased but only up to a certain concentration of 10 mg/L. All the four tested rice cultivars showed best response of callus induction at 10 mg/L. Super Basmati showed best callus induction frequency of 94% on 10 mg/L of CuO-NPs followed by Basmati 385 (90%), Basmati 370 (86%), and Basmati 2000 (74%). On increasing the concentration of CuO-NPs to 15 mg/L decrease in callus induction frequency is observed. Least callus induction frequencies were recorded on higher concentration (15–20 mg/L) of CuO-NPs with (38%) of Basmati 385 on 20 mg/mL. The callus induction frequency is retarded on 20 mg/L as compared to the control.

**Table 1:** **Effect of different concentration of CuO-NPs on N6 medium for frequency of callus induction**.

***O. sativa* L. varieties**	**Control**	**CuO-NPs (1 mg/L)**	**CuO-NPs (5 mg/L)**	**CuO-NPs (10 mg/L)**	**CuO-NPs (15 mg/L)**	**CuO-NPs (20 mg/L)**
Basmati 2000	27.00 ± 1.1b (46%)	34.00 ± 1.3e (68%)	24.00 ± 2.9b (48%)	39.00 ± 2.7b (78%)	23.00 ± 4.7a (54%)	21.00 ± 1.1a (42%)
Basmati 370	21.00 ± 3.3a (42%)	37.00 ± 3.1e (74%)	26.00 ± 2.4b (52%)	43.00 ± 3.9d (86%)	22.00 ± 3.7a (44%)	19.00 ± 1.3a (38%)
Basmati 385	31.00 ± 1.7d (62%)	40.00 ± 2.2e (86%)	32.00 ± 3.6d (64%)	45.00 ± 1.1d (90%)	36.00 ± 2.5d (72%)	19.00 ± 2.2a (38%)
Super Basmati	29.00 ± 2.2d (58%)	46.00 ± 2.2f (92%)	33.00 ± 4.1c (66%)	47.00 ± 1.4d (94%)	35.00 ± 1.1d (70%)	27.00 ± 2.8d (54%)

Alphabets (a,b,c,d,e,f) on values represents LSD at p < 0.05.

### Regeneration of callus with different concentrations of CuO-NPs on selected regeneration media

**Figure 6:**
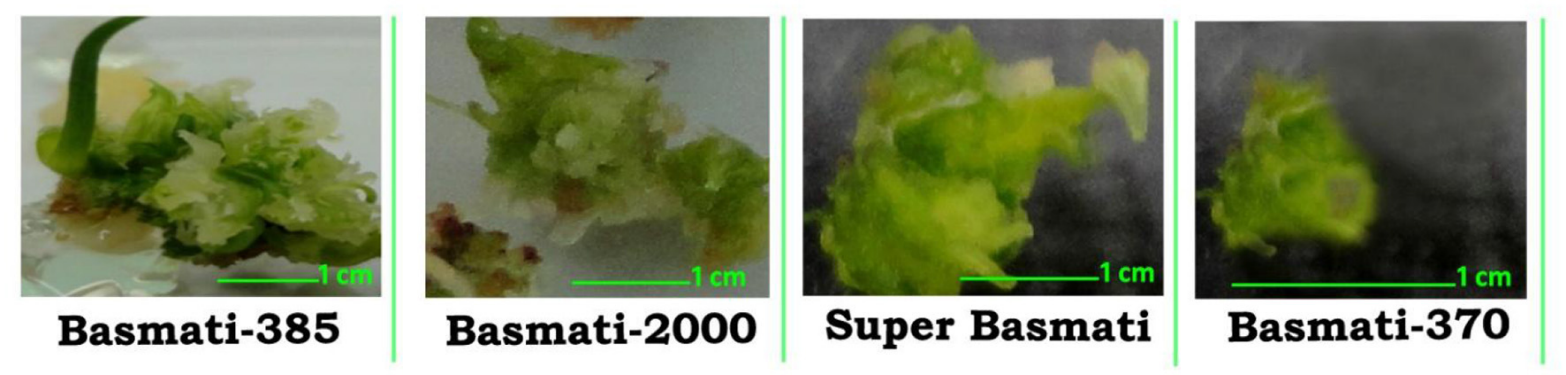
**Formation of green spot in ***O. sativa*** L. (Basmati 385, Basmati 2000, Super Basmati, and Basmati 370) calli after transferring to regeneration media**.

**Figure 7:**
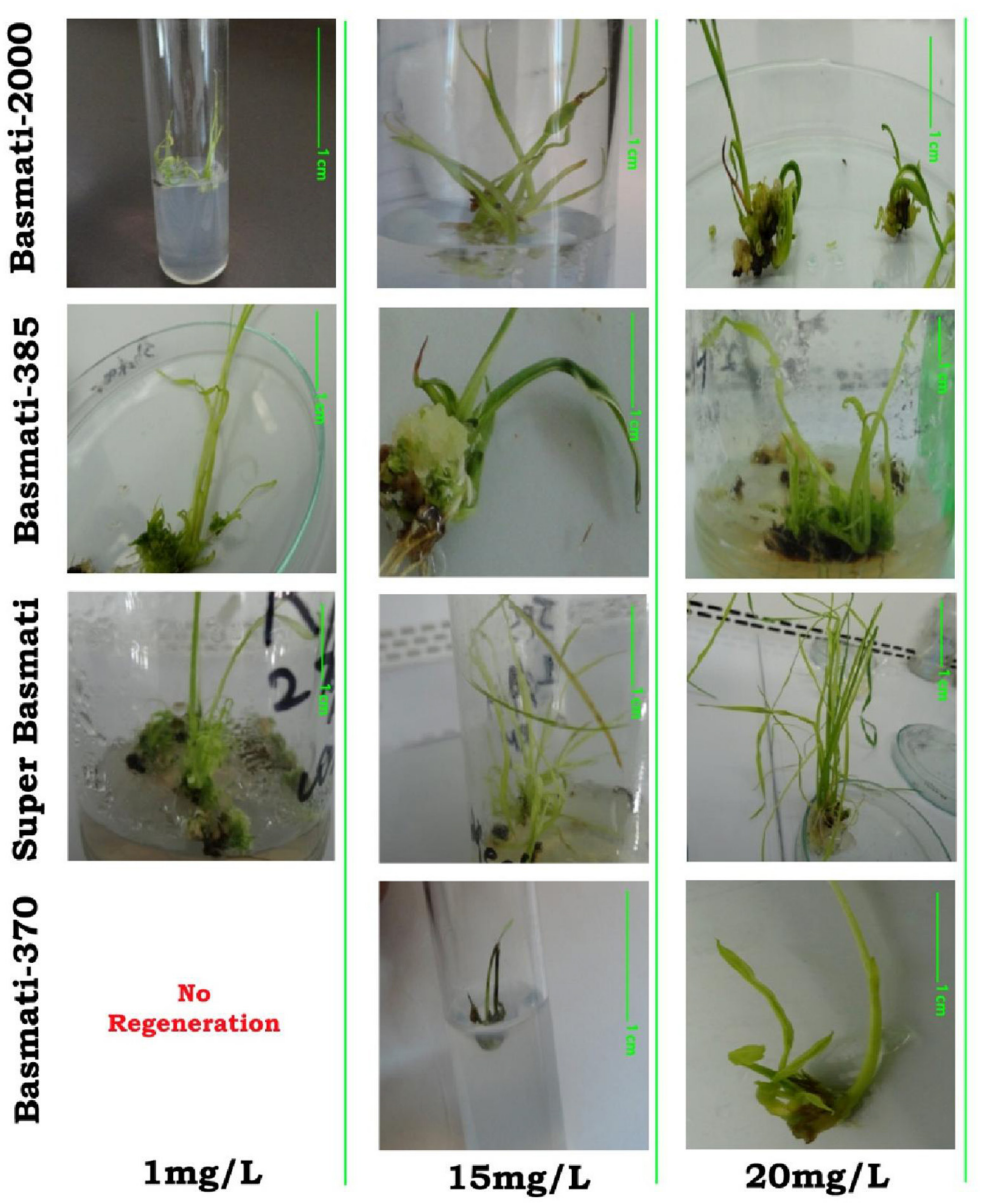
**Behavior of bio-synthesized CuO-NPs (1, 15, and 20 mg/L) in organogenesis of ***O. sativa*** L. (indeginous verities = Basmati 2000, Basmati 385, Super Basmati, and Basmati 370)**.

After the induction of calli the tested rice cultivars are shifted to regeneration media (NAA 1.0 mg/L + BAP 0.5 mg/L + Kin 0.5 mg/L) with various concentrations of CuO-NPs and compared with control. After shifting of calli to regeneration media green spots were formed in few days (Figure 6). The green spots were sub-cultured routinely to fresh media. The regeneration potential of all the rice cultivars were enhanced by the addition of CuO-NPs as compared to control and are presented in Table 2. In contrast to callogenesis the regeneration was best observed in Basmati 385 (92%) at higher concentration of 20 mg/L followed by Basmati 2000 (80%), Super Basmati (52%), and Basmati 370 (32%). Basmati 370 showed poor regeneration at lower CuO-NPs concentration as depicted in Figure 7. On supplementation of media with NPs concentration above 10 mg/L improved the organogenesis frequency. At 15 mg/L (37%) regeneration was reported as compared to control (20%) while best results were obtained on 20 mg/L of NPs for Basmati 370 as compared to control.

**Table 2:** **Effect of CuO-NPs on regeneration frequency**.

***O. sativa* L. varieties**	**Control**	**CuO-NPs (1 mg/L)**	**CuO-NPs (5 mg/L)**	**CuO-NPs (10 mg/L)**	**CuO-NPs (15 mg/L)**	**CuO-NPs (20 mg/L)**
Basmati 2000	17.00 ± 1.1c (42%)	27.00 ± 2.1d (67%)	21.00 ± 3c (52%)	16.21 ± 1.1b (40%)	27.00 ± 1.1 (67%)	32.33 ± 2.3e (80%)
Basmati 370	8.00 ± 1.3a (20%)	0.00 ± 0.00 (0%)	0.00 00 ± 0 (0%)	13.00 ± 2.3b (32%)	15.00 ± 1.0 (37%)	17.00 ± 3.1c (42%)
Basmati 385	24.00 ± 1.00d (60%)	33.67 ± 1.7e (82%)	23.00 ± 1.4d (57%)	19.00 ± 2.1c (47%)	24.00 ±2.2 (60%)	37.34 ± 1.0e (92%)
Super Basmati	15.33 ± 1.00c (37%)	19.00 ± 2.2c (47%)	11.00 ± 1.4b (27%)	9.00 ± 2.8a (22%)	26.00 ± 1.1d (65%)	21.00 ± 1.5c (52%)

Alphabets (a,b,c,d,e) on values represents LSD at p < 0.05.

## Discussion

### Characterization analysis of CuO-NPs

Homogenous spherical morphology of the synthesized NPs using SEM clearly hints the successful capping action of bio-active molecules from *A. indica* plant extract. All the peaks in XRD pattern are well indexed according to JCPDS Card No. 05-0661 which confirms the formation of single phase cubic fluorite structure. No peaks related to any impurity like Cu, Cu_2_O etc. is found. Stretching and vibrational mode of molecular structures in FTIR spectrum also provide sufficient data to investigate pure phase of prepared CuO-NPs sample. Cu-O exhibits its typical identification value on FTIR spectrum (Figure 4) confirming purely monoclinic morphology (Jan et al., 2014). Similarity in the specific spectrum of CuO-NPs spectrum was also observed previously (ThekkaePadil and Černík, 2013). Results from FTIR confirms XRD findings and clearly indicates formation of CuO-NPs via plant extract having pure phase structure.

### Study of CuO-NPs effect on callogenesis

The success of plant biotechnology depends on several factors which comprise an efficient tissue culture system for regeneration of plants from cultured cells and tissues (Khalafalla et al., 2010). Due to monoclinic structure, CuO-NPs have antimicrobial and antioxidant properties reported (Sivaraj et al., 2014). Present study revealed concentration dependent positive influence of CuO-NPs on callus induction in all the four tested cultivars. The best suited NPs concentration was recorded on 10 mg/L on N6 media supplemented with 2 mg/L 2,4-D. The positive effects of CuO-NPs on callus induction can be explain as copper is an essential nutrient in plants growth and act as a cofactor in many metallo-proteins and it also acts as structural element in regulatory proteins and involve in important physiological processes like electron transport chain, hormone signaling and cell wall metabolism (Yruela, 2005). With the increase in concentration the decrease in callogenesis was recorded, this could be best explain by the fact that high concentrations of NPs prove toxic for living system as release of Cu ions from CuO-NP induce oxidative stress by catalyzing the formation of (OH) radicals from non-enzymatic chemical reaction between superoxide and H_2_O_2_ which is proved toxic for living system (Cheloni et al., 2016). At cellular level Copper toxicity is due to the binding of Cu ions to sulfhydryl groups in proteins this in turn inhibit enzyme activity (Van Assche and Clijsters, 1990). Copper ions are readily release from CuO-NP and are highly impermeable to plasma membrane thus cause deficiency of other essential nutrients (Meharg, 1994).

### Regeneration of callus with different concentrations of CuO-NPs

The calli derived from all of the four tested cultivars were transferred to regeneration media control and with different concentrations of CuO-NPs to accesses the effects of NPs on regeneration potential. In contrast to calli induction with green spot formation, organogenesis was best reported on RM with 20 mg/L CuO-NPs as they interacted with plants and cause morphological and physiological changes (Khodakovskaya et al., 2013). The positive or negative effects of NPs primarily depends upon composition, size, and concentration (Ma et al., 2010). With the increase in concentration from 1 to 20 mg/L significant enhanced organogenesis was observed. Similar results with increase in concentrations of ZnO NPs increase shoot regeneration in banana plant was reported by Helaly et al. (2014). The improvement in regeneration with the addition of CuO-NPs can be explained through both mechanisms as Cu an essential nutrient in plants growth and effects many physiological processes positively. Cu triggers some enzymes in plants which are involved in lignin synthesis and it is vital in several enzyme systems. It is also required in the process of photosynthesis, is essential in plant respiration and assists in plant metabolism of carbohydrates and proteins. Cu helps to promote the production and creation of seeds (Alaoui-Sossé et al., 2004). Other mechanism through which NPs can improve regeneration is by increasing the total proline contents in cell. Joshi et al. (2011) reported that NPs induce stress conditions which in turn cause increased proline contents. Proline is a biochemical marker, produced in cultured cells, during stress, and high concentration can save the plants' cells from getting into unfavorable conditions.

It was mainly observed that NPs addition increase enzyme activity like peroxidase, catalase, and nitrate reductase activity which also favors regeneration through effecting important physiological and biochemical processes (Husen and Siddiqi, 2014). Moreover regenerated plantlets treated with different concentrations of NPs reported better chlorophyll a/b ratio. This ratio indicates the activeness of PS-I and PS-II defining the overall rate of photosynthesis as more photosynthesis will lead to more regeneration (Parida and Das, 2005). Enhancement of chlorophyll pigments by the supplementation of TiO_2_ was also reported by Zheng et al. (2005). CuO-NPs can be toxic at higher concentration as Cu is amongst the indispensable elements in maintaining homeostasis in living system so can cause genotoxicity so supplementation of CuO-NPs in tissue culture should not accede physiological tolerance range (Chang et al., 2012).

## Conclusion

Green chemistry is proved to be most reliable, eco-friendly and preferably biocompatible technique to synthesized extremely fine size CuO-NPs. Due to small size of 40 ± 5 nm with large surface area, effect of NPs on callus induction frequency concludes positive nutritive effect up to the concentration of 10 mg/L. We have experimentally proved that indigenous rice verity Super Basmati (*O. sativa* L.) shows maximum callogenesis response of 94%.In contrast to callus induction the organogenesis was best observed in Basmati 385 (*O. sativa* L.) of 92%.Due to strong antimicrobial behavior of CuO-NPs (Ren et al., 2009), it can also be an ideal candidate in plant tissue culture to ensure the environment aseptic. Keeping in view the importance of CuO-NPs as vital nutritive agent for normal plants growth, as along with its cytotoxic behavior, an optimized concentrations of CuO-NPs can be very beneficial in *in vitro* propagation of plants in future.

## Author contributions

All authors listed, have made considerable, direct and intellectual contribution to the work, and approved it for publication.

### Conflict of interest statement

The authors declare that the research was conducted in the absence of any commercial or financial relationships that could be construed as a potential conflict of interest. The reviewer TJ declared a shared affiliation, though no other collaboration, with several of the authors SA, NJ, FA to the handling Editor, who ensured that the process nevertheless met the standards of a fair and objective review.
