# Addendum: The Effect of Green Synthesized CuO Nanoparticles on Callogenesis and Regeneration of *Oryza sativa* L.

**DOI:** 10.3389/fpls.2020.00540

**Published:** 2020-05-06

**Authors:** Sadaf Anwaar, Qaisar Maqbool, Nyla Jabeen, Mudassar Nazar, Fazal Abbas, Bushra Nawaz, Talib Hussain, Syed Z. Hussain

**Affiliations:** ^1^Department of Biotechnology and Bioinformatics, International Islamic University Islamabad, Islamabad, Pakistan; ^2^Department of Biotechnology, Virtual University of Pakistan, Lahore, Pakistan; ^3^Interdisciplinary Research Organization, University of Chakwal, Chakwal, Pakistan; ^4^Department of Physics, International Islamic University Islamabad, Islamabad, Pakistan; ^5^Department of Mechatronics, University of Engineering and Technology Taxila, Chakwal, Pakistan; ^6^National Institute of Vacuum Science and Technology, Islamabad, Pakistan; ^7^Department of Biological Sciences, Quaid-i-Azam University, Islamabad, Pakistan

**Keywords:** copper oxide nanoparticles, SEM, XRD, FTIR, callus induction, regeneration

In the original article, the graph shown in FTIR spectrum analysis of bio-fabricated CuO-NPs (Figure 4) was plotted using a set of key data points (height vs. peaks) obtained from SHIMADZU™ FTIR scan, as shown in Figure 1 below. However, the FTIR plot (Figure 4) generated using data points alone without presenting the original SHIMADZU™ FTIR scan does not satisfy the plotting curve and complete information about FTIR analysis of CuO-NPs.

**Figure 1:**
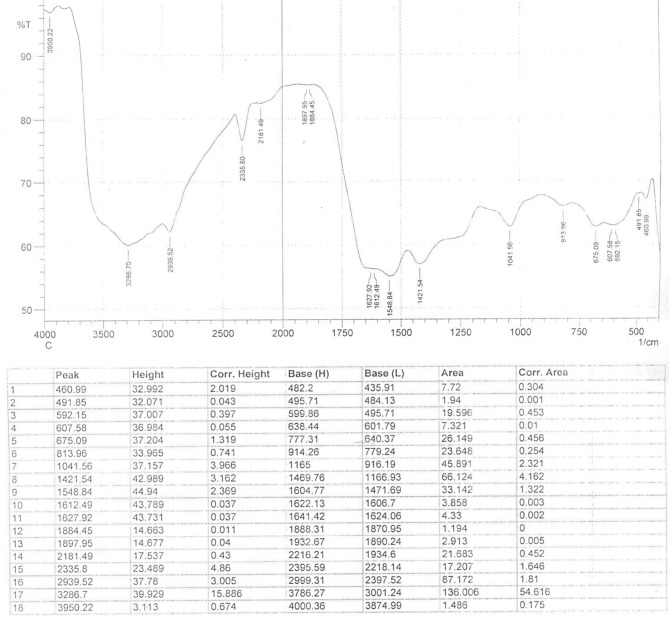
FTIR spectrum analysis of bio-fabricated CuO-NPs.

In order to provide a clear understanding of FTIR analysis of CuO-NPs, the FTIR scan showing the original FTIR plot as well as data points will be considered additionally, as shown in Figure 1 below. Furthermore, the underlining text about FTIR analysis in the Results section, subsection Characterization of Prepared CuO-NPs and the Discussion section, subsection Characterization Analysis of CuO-NPs will remained unchanged. In addition to this, and for the detailed understanding of the readers, the plotting video of FTIR spectrum (Figure 4) has been added as Supplementary Material.

